# Comprehensive analysis of HDAC7 expression and its prognostic value in diffuse large B cell lymphoma: A review

**DOI:** 10.1097/MD.0000000000034577

**Published:** 2023-11-10

**Authors:** Weiguo Lu, Guangyan Zhuang, Youmin Guan, Yongcong Li, Liujun Liu, Mingfeng Xiao

**Affiliations:** a The First Affiliated Hospital of Guangzhou University of Chinese Medicine, Guangzhou, China; b Guangzhou University of Chinese Medicine, Guangzhou, China.

**Keywords:** biomarker, diffuse large B cell lymphoma, HDAC7, immune infiltration, prognosis

## Abstract

HDAC7 loss or dysregulation may lead to B cell-based hematological malignancies. This study aimed to explore the prognostic value of HDAC7 in patients with diffuse large B cell lymphoma (DLBCL). RNA sequencing data and clinical information for HDAC7 in DLBCL were collected from the cancer genome atlas database and analyzed using R software. Paired *t* and Mann–Whitney *U* tests were used to detect differences between DLBCL and adjacent normal tissues, and the pROC software package was used to generate receiver operator characteristic curves to detect cutoff values for HDAC7. Data from paraffin-embedded specimens from the 2 groups were used for validation of external immunohistochemical staining. The tumor immunity estimation resource and integrated repository portal for tumor immune system interactions databases were used to analyze the correlation between HDAC7 and DLBCL immune cell infiltration. Survival analysis of HDAC7 in patients with DLBCL was performed using the PrognoScan database. Compared with that in normal tissues, HDAC7 mRNA was overexpressed in DLBCL. The HDAC7 immunohistochemical staining scores of stage III and IV DLBCL patients were significantly lower than those of stage I and II DLBCL patients, which was associated with shorter overall survival and disease-specific survival. In addition, the higher expression of HDAC7 may play a role in the lower level of immune infiltration in DLBCL. Downregulation of HDAC7 expression was correlated with poor prognosis and immune infiltration in DLBCL patients.

## 1. Introduction

Diffuse large B cell lymphoma (DLBCL) is the most common non-Hodgkin lymphoma, accounting for approximately 30% to 40% of all cases in different regions.^[1]^ Most patients present with a rapidly growing lymphoma mass, often involving 1 or more lymph nodes and extranodal sites.^[2]^ Extranodal tumors are present in up to 40% of DLBCL patients. The primary site of DLBCL can be any tissue or organ, with the gastrointestinal tract being the most common.^[1]^ The standard chemotherapy regimen for DLBCL patients is rituximab, cyclophosphamide, doxorubicin, vincristine, and prednisone (R-CHOP). Approximately 60% to 70% of DLBCL patients can be treated with this regimen. However, a small percentage of patients do not respond to R-CHOP, and approximately 30% to 40% relapse.^[3]^ The treatment options are limited for this subset of patients. In addition, the tumor activity of DLBCL varies greatly from indolent to highly lethal, which can lead to very different treatment outcomes.^[4]^ Therefore, it is important to understand DLBCL tumor biology and explore prognostic biomarkers for DLBCL patients.

Members of the class IIa histone deacetylase (HDAC) subfamily are tissue-specific gene repressors that play important roles in development and differentiation. HDAC7 is a class IIa HDAC that exhibits a lymph-specific expression pattern in the hematopoietic system.^[5]^ DLBCL tumor cells express pan-B cell antigens such as CD19, CD20, CD22, and other B cell transcription factors. According to previous studies, loss of HDAC7 dramatically stunts early B cell development and causes severe lymphocytopenia in peripheral organs as well as confusion in the lineage of pro-B cells.^[6]^ These findings shed light on the mechanisms by which HDAC7 loss or dysregulation may lead to B cell-based hematological malignancies.^[7]^ However, the predictive value of HDAC7 in DLBCL in clinical practice has not yet been explored. Therefore, we used data from the cancer genome atlas (TCGA) to explore its potential role in DLBCL.

With the development of high-throughput sequencing and microarray technologies, we can identify key genes related to tumor prognosis and progression using bioinformatic analysis. We identified several potential biomarkers of DLBCL based on TCGA data mining. After searching PubMed (https://pubmed.ncbi.nlm.nih.gov/), we found no studies revealing the role of HDAC7 in DLBCL. Therefore, this study aimed to investigate the prognostic role of HDAC7 in DLBCL and its association with immunity.

## 2. Methods

### 2.1. Data acquisition and processing

We downloaded data from the official website of TCGA (https://portal.gdc.cancer.gov/) for several cancers, including DLBCL HDAC7 transcriptome data and related clinical information.^[8]^ The normal group had at least 5 samples for each of the 30 cancers included. The initially FPKM-formatted data were converted to TPM and log2 formats for further study. Finally, the RNA-seq data of 444 DLBCL and 47 adjacent normal tissues were retained. All selected samples included HDAC7 gene expression data and relevant clinical information, including age, sex, clinical stage, and survival status.

### 2.2. Expression analysis of HDAC7

The mRNA expression data are presented as mean ± SD. We used R software (v3.6.3) (https://www.r-project.org/) to analyze all statistics and visualize the differences using the ggplot2 package. Differences between DLBCL and adjacent normal tissues were examined using the paired *t* and Mann–Whitney *U* tests. A receiver operator characteristic (ROC) curve was generated to detect the cutoff of HDAC7 using the pROC software package.^[9]^ In this study, UALCAN (https://ualcan.path.uab.edu/) was used to comprehensively analyze HDAC7 protein expression.^[10]^

### 2.3. Immunohistochemical staining and evaluation

DLBCL tissues from the 2 groups were stained by immunohistochemistry (IHC) to detection the protein expression level of HDAC7. One group consisted of 10 paraffin-embedded DLBCL samples from stage I and II patients, whereas the other group consisted of 10 stage III and IV patients. All tissues were obtained from The First Affiliated Hospital of Guangzhou University of Chinese Medicine. The state of the tissues was confirmed by 2 pathologists after hematoxylin and eosin staining.

IHC staining was performed on the tissue microarray slides and paraffin sections. These were then incubated with an anti-HDAC7 primary antibody (Affinity, Changzhou, China, 50 uL) and a secondary antibody (Rabbit Anti-Goat IgG H&L (HRP), Servicebio, Wuhan, China, 100 uL). Staining was visualized with DAB solution and hematoxylin counterstaining. Images were acquired using a microscope (Olympus BX43) at x200. The average optical density (MOD) of HDAC7 in the tumor tissue was calculated using the immunohistochemical staining method combined with GraphPad Prism software (San Diego, CA). The results were analyzed using means and compared using the 2-tailed unpaired Student *t* test. If the data were not normally distributed (assessed using D’Agostino-Pearson omnibus normality test), the samples were analyzed using Mann–Whitney nonparametric test.

### 2.4. Univariate/multivariate COX hazard regression analyses and nomogram construction

Univariate/multivariate COX hazard regression was used to analyze the independent prognostic factors and clinical characteristics of DLBCL (age, grade, stage, sex, race, treatment outcome, etc.) using R (version 3.5.1; https://www.r-project.org/). To predict the probability of overall survival (OS), the R “RMS” and “Survival ROC” packages were used to create a nomogram, and the area under the curve (AUC) between individual predictors and survival was calculated. Calibration curves and C-indices were used to evaluate the performance of the constructed nomogram.

### 2.5. Protein-protein interaction and enrichment analysis of HDAC7

A protein-protein interaction (PPI) network was constructed using the public database STRING (http://string-db.org) to retrieve the co-expressed genes. Functionally condensed analyses of co-expressed genes were performed using the “Cluster Profiler” R package and visualized using the “ggplot2” R package in Gene Ontology and the Kyoto Encyclopedia of Genes and Genomes.^[11,12]^

### 2.6. Tumor immune infiltration, immune checkpoint molecules, and immune cell pathways

Based on the the tumor immunity estimation resource database, the relationship between HDAC7 expression in DLBCL and 6 types of immune-infiltrating cells, including B cells, CD4^+^ T cells, CD8^+^ T cells, neutrophils, macrophages, and dendritic cells, was analyzed. Tumor immune system interactions (http://cis.hku.hk/TISIDB/) was used to explore HDAC7 expression in tumor tissues and tumor-infiltrating lymphocytes (TILs).^[13]^ After analyzing the gene expression profiles, gene set variation analysis was used to determine the relative abundance of the TILs. We also calculated immune cell infiltration in DLBCL using CIBERSORT with *P* < .001 as the cutoff value.^[14]^ We conducted these analyses using the “limma” R package and visualized this using the “reshape2” and “RColorBrewer” R packages. The aforementioned analyses were performed using the Sangerbox tools (http://www.sangerbox.com/tool).

### 2.7. Ethics approval and consent to participate

The present study was approved by The First Affiliated Hospital of Guangzhou University of Chinese Medicine and was conducted in accordance with the Helsinki Declaration of 1964 (revised in 2013).

## 3. Results

### 3.1. Elevated expression of HDAC7 from a pan-cancer perspective

**Figure 1.**
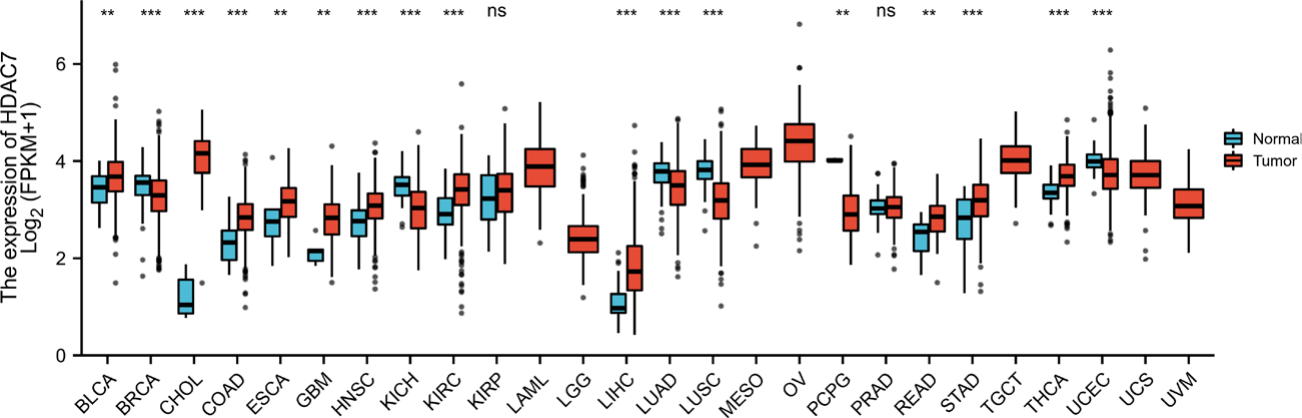
Expression pattern of HDAC7 from a pan-cancer perspective. *P < .05; ***P < .001. BLCA = bladder urothelial carcinoma, BRCA = breast invasive carcinoma, CHOL = cholangiocarcinoma, COAD = colon adenocarcinoma, ESCA = esophageal carcinoma, GBM = glioblastoma mutiforme, HNSC = head and neck squamous cell carcinoma, KICH = kidney chromophobe, KIRC = kidney renal clear cell carcinoma, KIRP = kidney renal papillary cell carcinoma, LIHC = liver hepatocellular carcinoma, LUAD = lung adenocarcinoma, LUSC = lung squamous cell carcinoma, PRAD = prostate adenocarcinoma, PCPG pheochromocytoma and paraganglioma, READ = rectum adenocarcinoma, STAD = stomach adenocarcinoma, THCA = thyroid carcinoma, UCEC = uterine corpus endometrial carcinoma, USC = uterine serous carcinoma, UVM = uveal melanoma.

To accurately assess the expression pattern of HDAC7 mRNA in different tumors, datasets containing 7 cancer types with fewer than 5 samples in the normal group were excluded from the analysis. Finally, as shown in Figure 1, the expression levels of HDAC7 mRNA in all carcinomas (26 cancers) were obtained from TCGA. These data demonstrated that HDAC7 mRNA is abnormally expressed in different cancer types.

### 3.2. Relative expression level of HDAC7 in DLBCL

**Figure 2.**
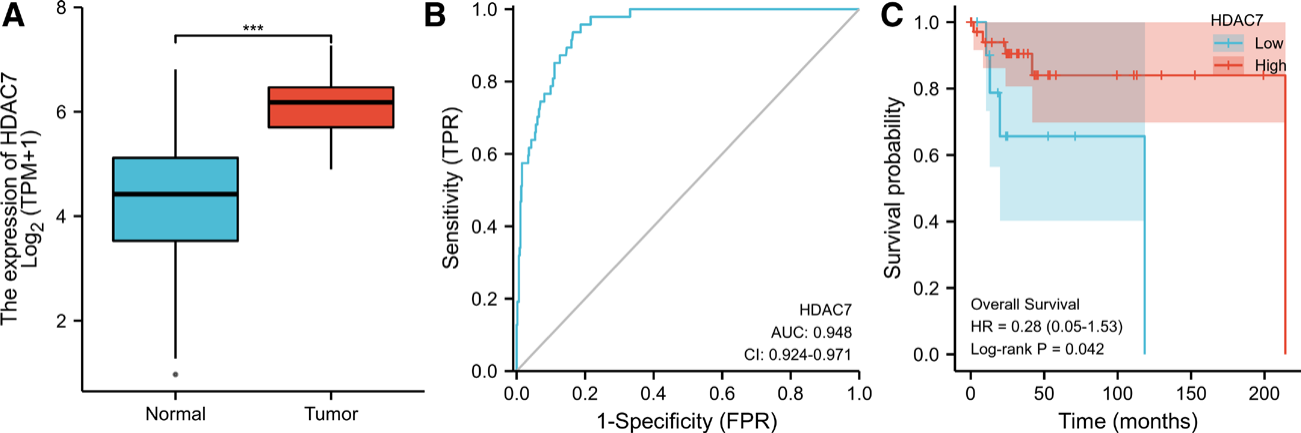
Relative expression level and survival analysis of HDAC7 in DLBCL based on TCGA database, (A) boxplot of HDAC7 expression between diffuse large B cell lymphoma (DLBCL) and normal tissues in the cancer genome atlas (TCGA) dataset (normal = 47 and tumor = 444), (B) receiver operator characteristic (ROC) curves and their area under the curve (AUC) for HDAC7, (C) Kaplan–Meier (K–M) survival analysis of HDAC7. *P < .05; ***P < .001.

We determined the mRNA and protein expression levels of HDAC7 in DLBCL after analyzing the expression data of HDAC7 in TCGA. The expression level of HDAC7 mRNA in DLBCL tissues (n = 444) was significantly higher than that in adjacent normal tissues (n = 47) (4.283 ± 1.082 vs 6.180 ± 0.765, *P* < .001), as shown in Figure 2A. These results indicate that mRNA of HDAC7 expression is higher in DLBCL tissues. ROC curve analysis showed that the AUC value of HDAC7 was 0.948 (95% CI: 0.924–0.971) (Fig. 2B). When the cutoff value was 5.359, the sensitivity, specificity, and accuracy of HDAC7 were 93.6%, 83.6%, and 77.2%, respectively. The positive and negative predictive values were 37.6% and 99.2%, respectively. Our analysis showed that HDAC7 is a promising biomarker for distinguishing DLBCL tissues from normal tissues. Kaplan–Meier survival analysis showed that DLBCL patients in the low-HDAC7 groups had a significantly decreased OS compared with those in the high-HDAC7 groups (*P* = .042, Fig. 2C).

### 3.3. Protein expression of HDAC7 in two groups of tissue samples

**Figure 3.**
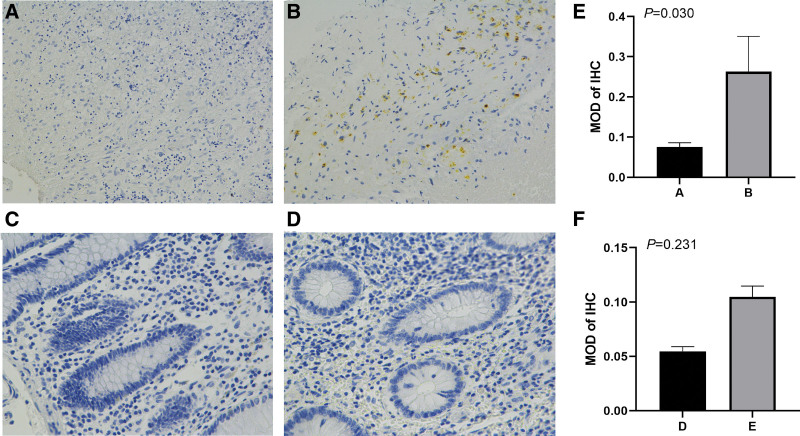
Protein expression level of HDAC7 in tissues from different groups of diffuse large B cell lymphoma (DLBCL) patients. (A) Expressions of HDAC7 in the DLBCL tissues of subclavian lymph nodes from patients with stages IV (×200). (B) Expressions of HDAC7 in the DLBCL tissues of subclavian lymph nodes from patients with stages I (×200). (D) Expressions of HDAC7 in the DLBCL tissues of cecum from patients with stages IV (×200). (E) Expressions of HDAC7 in the DLBCL tissues of cecum from patients with stages II (×200). (C and F) Comparation of MOD of the IHC of DLBCL tissues from group A and B, group D and E, respectively. IHC = immunohistochemical staining.

IHC staining was performed on DLBCL tissues from different groups (stages I and II vs stages III and IV) to validate the differential expression of HDAC7 at the protein level. These images were representative of tissues obtained from subclavian lymph nodes and cecum of patients with DLBCL. As shown in Figure 3, representative stained spots were mainly observed in the cytoplasm of cells, and a lower expression of HDAC7 was observed in stage III and IV DLBCL tissues compared to stage I and II tissues. Specifically, as shown in Figure 3C, the MOD of HDAC7 for Figure 3A and Figure 3B were 0.080 ± 0.006 and 0.270 ± 0.022, respectively (*P* = .03). As shown in Figure 3F, The MOD for Figure 3D and Figure 3E were 0.024 ± 0.003 and 0.104 ± 0.002 respectively (*P* = .003). These results confirmed that HDAC7 is upregulated at the protein level in early-stage DLBCL patients.

### 3.4. Relationships between HDAC7 mRNA levels and clinical pathological characteristics of DLBCL patients

**Figure 4.**
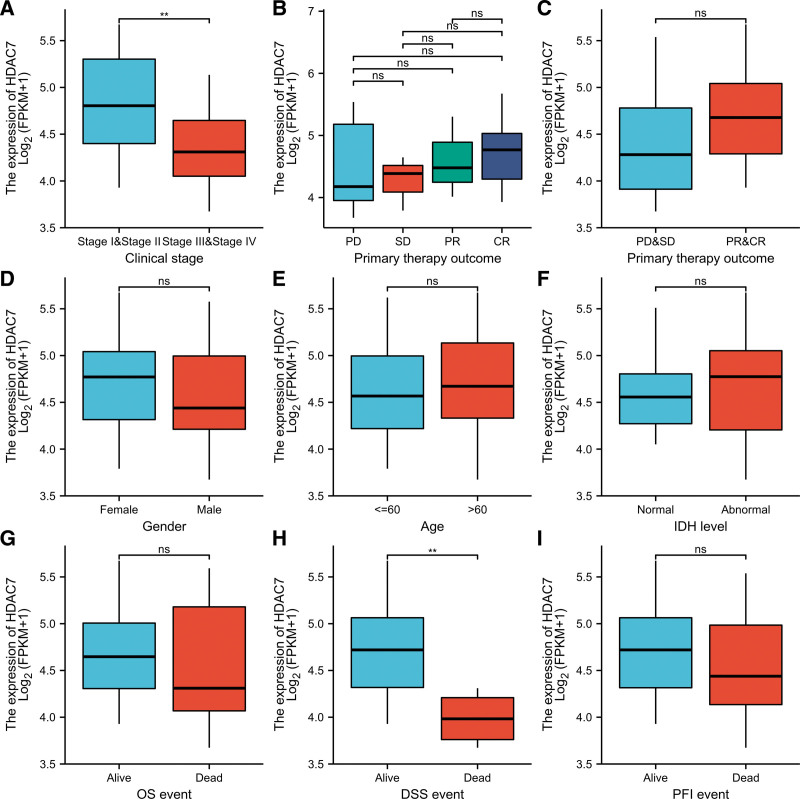
Relationships between HDAC7 mRNA levels and clinical pathological characteristics.

Table 1 shows the baseline characteristics of patients with DLBCL. The Mann–Whitney *U* test and logistic regression were used to analyze the relationship between HDAC7 mRNA expression and clinicopathological features according to TCGA data. As shown in Table 1 and Figure 4A–I, the expression level of HDAC7 was significantly correlated with clinical stage (stages I and II VS stages III and IV, *P* = .003). As shown in Figure 4H, the disease-specific survival (DSS) of DLBCL patients with high HDAC7 expression was significantly longer than those with low HDAC7 expression (*P* = .006). However, we detected no statistically significant relationship between the expression levels of HDAC7 and other clinical pathological features, such as primary therapy outcome (*P* = 1.000), sex (*P* = .385), isocitrate dehydrogenase (IDH) level (*P* = .462), and OS (*P* = 1.000). In summary, these results suggest that HDAC71 is highly correlated with earlier tumor clinical staging and longer survival time, further suggesting that HDAC7 may be a biomarker for better prognosis in DLBCL patients.

**Table 1:** Clinical characteristics of the diffuse large B cell lymphoma patients (TCGA).

Characteristic	Low expression of HDAC7	High expression of HDAC7	*P* value
n	24	24	
Clinical stage, n (%)			.054
Stage I	2 (4.8%)	6 (14.3%)	
Stage II	8 (19%)	9 (21.4%)	
Stage III	2 (4.8%)	3 (7.1%)	
Stage IV	10 (23.8%)	2 (4.8%)	
Primary therapy outcome, n (%)			1.000
PD	3 (6.5%)	2 (4.3%)	
SD	2 (4.3%)	1 (2.2%)	
PR	2 (4.3%)	1 (2.2%)	
CR	17 (37%)	18 (39.1%)	
Gender, n (%)			.385
Female	11 (22.9%)	15 (31.2%)	
Male	13 (27.1%)	9 (18.8%)	
Age, n (%)			.561
<=60	15 (31.2%)	12 (25%)	
>60	9 (18.8%)	12 (25%)	
IDH level, n (%)			.462
Normal	8 (26.7%)	5 (16.7%)	
Abnormal	7 (23.3%)	10 (33.3%)	
OS event, n (%)			1.000
Alive	19 (39.6%)	20 (41.7%)	
Dead	5 (10.4%)	4 (8.3%)	
DSS event, n (%)			.109
Alive	20 (41.7%)	24 (50%)	
Dead	4 (8.3%)	0 (0%)	
PFI event, n (%)			.739
Alive	17 (35.4%)	19 (39.6%)	
Dead	7 (14.6%)	5 (10.4%)	
Age, mean ± SD	53.17 ± 14.08	59.38 ± 13.38	.124

CR = complete response, DSS = disease-specific survival, IDH = isocitrate dehydrogenase, OS = overall survival, PD = progressive disease, PFS = progression Free Survival, PR = partial response, SD = stable disease, TCGA = the cancer genome atlas.

Additionally, Cox univariate and multivariate survival analysis showed that high expression of HDAC7 (*P* = .023) combined with response therapy outcome (*P* = .006) was correlated with longer DSS in DLBCL patients (Table 2), confirming our previous result. These results demonstrate that HDAC7 expression can be used to guide clinical work and has high value in evaluating the efficacy of treatment in patients with DLBCL.

**Table 2:** Cox regression analysis of clinical prognosis.

Characteristics	Total (N)	HR (95% CI)		*P* value	
		Univariate analysis	Multivariate analysis	Univariate analysis	Multivariate analysis
Clinical stage (stage I & stage II vs stage III & stage IV)	42	4.399 (0.448–43.201)		.204	
Primary therapy outcome (PD & SD vs PR & CR)	46	0.041 (0.004–0.394)	0.178 (0.012–2.713)	.006*	.214
Gender (female vs male)	48	0.427 (0.044–4.108)		.461	
Age (<=60 vs > 60)	48	0.608 (0.063–5.847)		.667	
HDAC7	48	0.003 (0.000–0.447)	0.029 (0.000–4.321)	.023*	.165

CR = complete response, PD = progressive disease, PR = partial response, SD = stable disease.

* *P* < .05.

### 3.5. Nomogram construction based on HDAC7 and clinicopathologic variables

**Figure 5.**
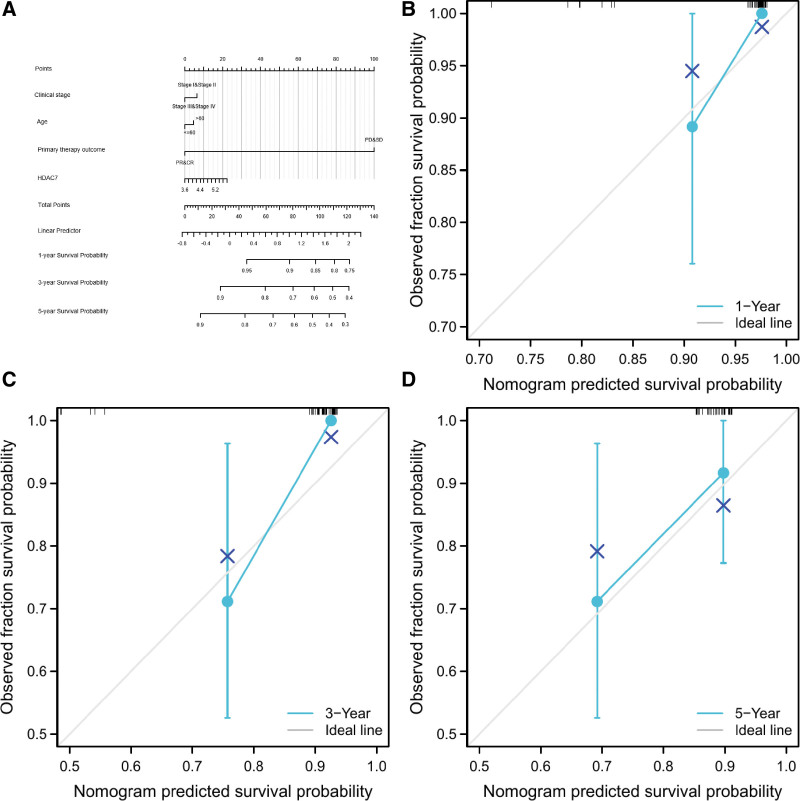
Nomogram construction and evaluation, (A) nomogram construction based on HDAC7 and clinicopathologic variables, (B) calibration curves of 1-, (C) 3-, (D) and 5-year survival.

Based on our nomogram construction, we estimated the survival rates of DLBCL patients at 1, 3, and 5 years, and obtained the total points. Thus, we concluded that the predictive method is more intuitive (Fig. 5A). The calibration curves of 1, 3, and 5-year survival (Fig. 5B–D) further showed that our nomograph construction was accurate.

### 3.6. PPI network and functional annotations

**Figure 6.**
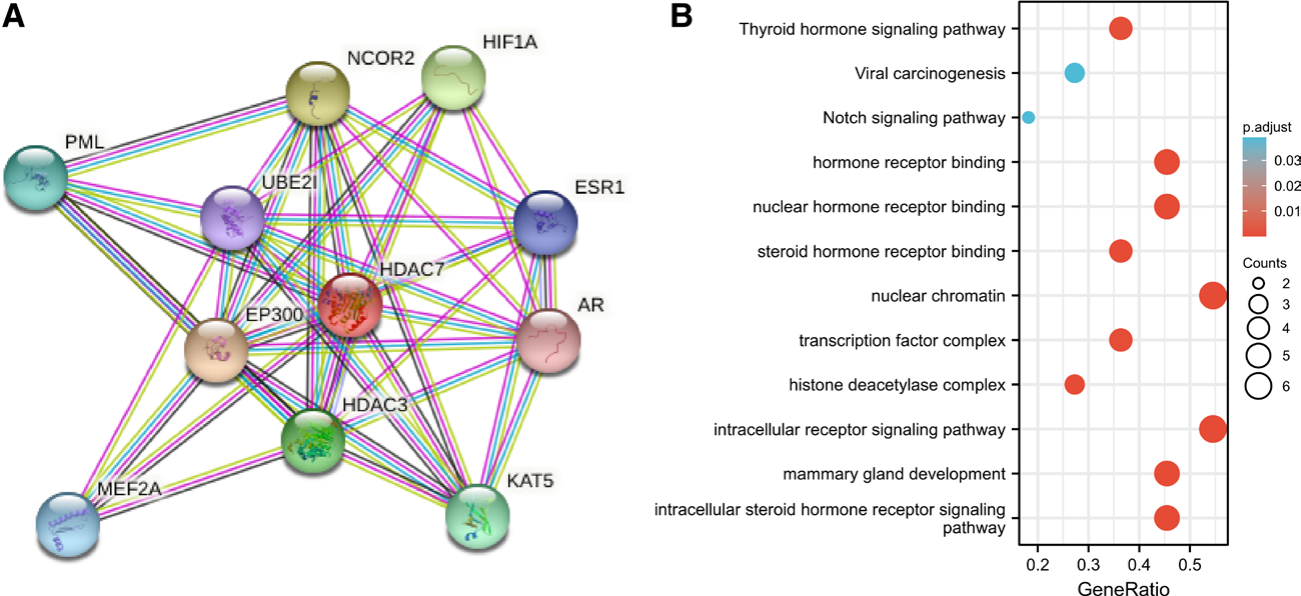
(A) PPI networks and functional enrichment analyses, (B) network of HDAC7 and its co-expressed genes. PPI = protein-protein interaction.

We constructed PPI networks and functional annotations based on the Gene Ontology string database and the Kyoto Encyclopedia of Genes and Genomes. Figure 6A shows HDAC7 and its network of ten co-expressed genes. Our results indicate that changes in the biological processes of HDAC7 are related to nuclear chromatin and intracellular receptor signaling pathways. Functional annotation indicated that these genes were involved in the Notch signaling pathway (Fig. 6B).

### 3.7. Relationships between HDAC7 and tumor microenvironment, tumor immune infiltration, immune cell pathways, and immune checkpoint molecules in DLBCL

**Figure 7.**
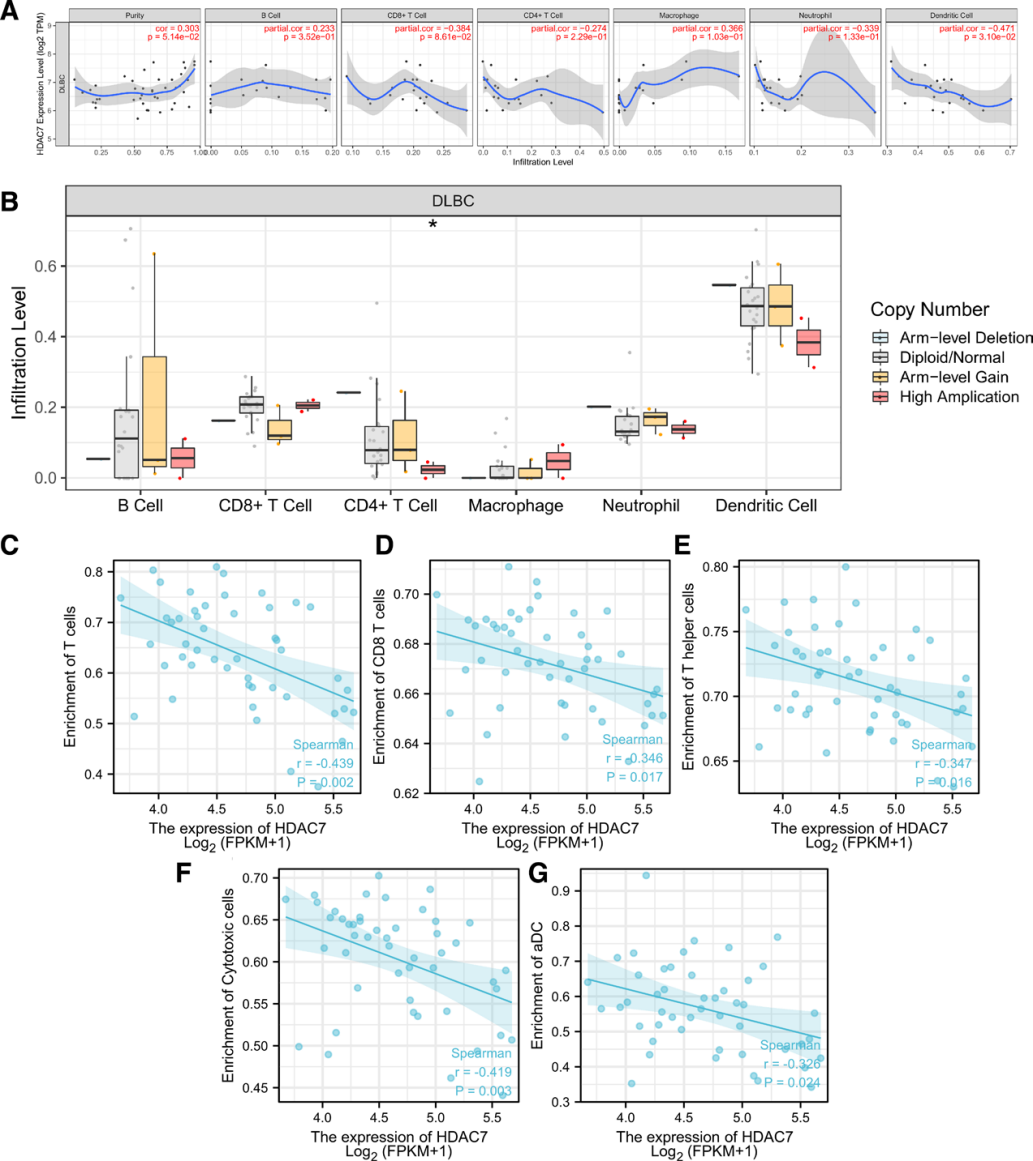
Association between HDAC7 expression and tumor immune infiltration in diffuse large B cell lymphoma (DLBCL). (A) HDAC7 expression is negatively correlated with dendritic cell infiltration in DLBCL, (B) associations between HDAC7 expression and SCNA in DLBCL, (C–G) association between HDAC7 expression and tumor immune infiltration in DLBCL. *P < .05.

**Figure 8.**
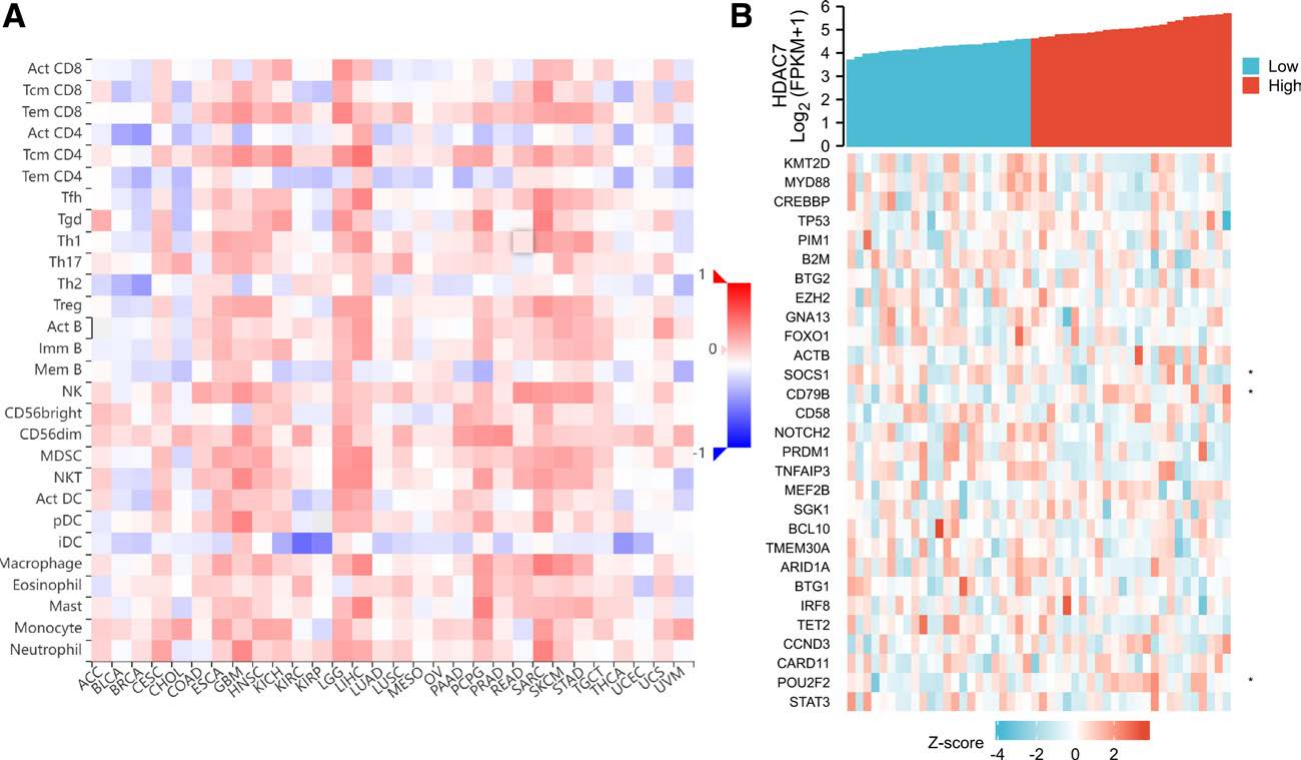
Relationships between HDAC7 and immune checkpoint molecules and immune cell pathways in diffuse large B cell lymphoma (DLBCL). (A) Co-expression analysis of HDAC7 and immune cell pathways in 28 types of cancer, (B) Co-expression analysis of HDAC7 and immune checkpoint molecules in DLBCL.

We analyzed tumor immune infiltration, immune cell pathways, and immune checkpoint molecules to further explore the potential relationship between HDAC7 and immunity (Figs. 7–8). As shown in Figure 7A, high levels of HDAC7 were significantly associated with downregulation of dendritic cell (r = −0.471, *P* = .031), cytotoxic cells (r = −0.419, *P* = .003), aDC (r = −0.323, *P* = .024), and T cell infiltration (r = −0.439, *P* = .002), including CD4^+^ T cells (r = −0.346, *P* = .017) and T helper cells (r = −0.347, *P* = .016). Using the Wilcoxon rank sum test, we used the SCNA module to explore the correlation between the level of tumor immune infiltration in DLBCL and different somatic copy number changes in HDAC7 (Fig. 7B). The association between HDAC7 expression and tumor immune infiltration in DLBCL is shown in Figure 7C–G. As shown in Figure 8A, considering the immune cell pathways, we propose an association between the expression of HDAC7 mRNA and 28 types of TILs in human cancer. Co-expression analysis between HDAC7 and immune checkpoint molecules indicated that HDAC7 was markedly related to B2M, ACTB, CD79B, BTG1, TET2, and POU2F2 in DLBCL using TCGA (all *P* < .05, Fig. 8B).

## 4. Discussion

As an indispensable part of precision medicine in cancer, biomarkers are important for predicting cancer prognosis and treatment effects.^[15,16]^ Various prognostic biomarkers and prognostic markers have been identified and established in previous research.^[17,18]^

HDACs are indispensable transcriptional repressors involved in many physiological and pathological processes. Among these proteins, the class IIa HDAC subfamily, which includes HDAC4, HDAC5, HDAC7, and HDAC9, shares the specific characteristics with all other HDACs, such as interaction with transcription factors and tissue specificity.^[5]^ Among them, HDAC7 seems to act as a lymphoid-specific transcription inhibitor of various HDACs.^[19]^ Here, we found that HDAC7 can be used as a biomarker for better prognosis and longer survival in patients with DLBCL.

In this study, through TCGA data mining, we revealed the relationship between HDAC7 expression and survival of patients with DLBCL. Our results showed that HDAC7 expression was higher in DLBCL tumors than in normal lymph node tissues. However, in an internal comparison between patients with tumors, we found that HDAC7 expression was significantly higher in patients with stage I and II disease than in those with stage III and IV disease. Our IHC staining results, which validated the protein expression of HDAC7, were consistent with the above analysis. We found that elevated HDAC7 expression was associated with better OS and PFS with moderate diagnostic accuracy. Moreover, logistic regression analysis indicated that HDAC7 was significantly associated with DSS, PFS, and better primary therapy outcomes (complete and partial responses). Univariate/multivariate Cox hazard regression analysis also indicated that HDAC7 could act as an independent prognostic factor for DLBCL. In conclusion, these results suggest that HDAC7 may act as a prognostic predictor of survival in patients with DLBCL.

As predictive tools, nomograms have been widely used to assist in clinical decision-making.^[20,21]^ In this study, we created a nomogram using HDAC7 and 4 clinical parameters (age, grade, clinical stage, main treatment outcome, and HDAC7 expression) to predict the OS of patients with DLBCL. The C-index; 1, 3, and 5-year AUCs; and calibration curves indicated that this nomogram had a moderate prediction accuracy and satisfactory performance. Overall, we successfully established an HDAC7-based nomogram that is anticipated to guide the prognosis of patients with DLBCL.

To identify HDAC7-related signaling pathways and immunity, we further explored the potential relationships between HDAC7 and immunity, focusing on 3 aspects: tumor immune infiltration, immune cell pathways, and immune checkpoint molecules. As reported in previous studies, the tumor microenvironment and tumor immune infiltration are associated with DLBCL prognosis and response to immunotherapy, and various genes are significantly associated with immune cell pathways and checkpoint molecules.^[22,23]^ According to a previous study, naïve B cells, gamma delta T cells, and resting NK cells are the 3 most affected immune cells that influence several steps in the DLBCL immunity cycle.^[24]^ In our research, regarding tumor immune infiltration, high levels of HDAC7 were significantly associated with downregulated infiltration of dendritic, cytotoxic, aDC, and T cells. Co-expression analysis showed that HDAC7 was markedly related to B2M, ACTB, CD79B, BTG1, TET2, and POU2F2 in DLBCL. Overall, our study showed that HDAC7 may be closely related to immunity in DLBCL.

The relationship between gene expression and tumor metastasis and development has attracted attention. Over the years, with the help of single gene assessment methods, it has been found that gene mutation or expression cannot be ignored in the pathogenesis of DLBCL.^[25]^ Many cellular processes and pathways are involved in gene mutations in DLBCL, including histone modification (methylation and acetylation), cell growth, proliferation, metabolism, differentiation, apoptosis, survival, homing/migration, response to DNA damage, B-cell receptor signaling, Toll-like receptor signaling, angiogenesis, and immunoregulation.^[26–28]^ Through gene expression, we can understand the pathogenesis of the disease and, at the same time, use this as a potential target of prognostic value. The proper development of B lymphocytes depends on a genetic program unique to each cell stage that is based on appropriate transcriptional control. When these specific transcriptional controls are relaxed, B cell maturation is impeded, which is likely to lead to the development of hematological malignancies, such as leukemia and lymphoma. Moreover, HDAC7 expression plays an important role in inducing apoptosis and inhibiting the oncogenic potential of the cell lines tested in vitro and in vivo. In contrast, genome-wide transcriptome profiling showed that ectopically expressed HDAC7 induces the expression of apoptotic genes and leads to the downregulation of key oncogenes, such as c-Myc.^[29]^ DLBCL patients with MYC translocations at the immunoglobulin gene locus have a worse prognosis than those with a non-immunoglobulin gene partner.^[30]^ In some studies, HDAC7 expression was lower in samples from patients with B lymphocytes with a high Myc expression.^[29]^ The results from our study agree with this assumption and show that high expression of HDAC7 is associated with a lower degree of immune infiltration, which may play a role in inhibiting oncogenic potential. Further studies are required to confirm this association.

Our analysis shares some common limitations with similar studies. First, owing to the retrospective nature of the study, clinical information from TCGA was limited, and some important data could not be obtained. Second, our IHC staining verification results indicated that HDAC7 protein displayed relatively low expression in DLBCL tumors compared to the findings of previous studies. This was likely due to the limited number of clinical samples. Therefore, subsequent experiments should be performed using more samples to verify this expression. Moreover, we verified the potential mechanisms of HDAC7 in DLBCL at both cellular and molecular levels. Notwithstanding these limitations, our study had several strengths. To our knowledge, this is the first study to explore the role of HDAC7 in DLBCL. In addition, multiple elements were analyzed for HDAC7 in DLBCL, including mutation features, nomogram, PPI, tumor immune infiltration, immune cell pathways, and checkpoint molecules.

Recent advances in understanding the molecular discoveries in DLBCL can help in the development of novel and highly effective therapies for patients with DLBCL. Thus, we are fulfilling the potential of precision medicine. It is our hope that we can use the knowledge gained to develop innovative therapeutics.^[1]^

## 5. Conclusion

In summary, our study demonstrated that decreased HDAC7 expression was correlated with poor prognosis and immune infiltration in DLBCL, which may provide crucial information for the development of novel immunotherapies.

## Acknowledgements

We would like to thank Editage for the language editing provided for this manuscript.

## Author contributions

**Conceptualization:** Mingfeng Xiao.

**Data curation:** Weiguo Lu.

**Formal analysis:** Liujun Liu.

**Methodology:** Weiguo Lu.

**Resources:** Guangyan Zhuang.

**Supervision:** Yongcong Li.

**Writing – original draft:** Mingfeng Xiao.

**Writing – review & editing:** Youmin Guan.
